# Effectiveness and safety of acupuncture and moxibustion for chronic prostatitis: A protocol for an overview of systematic reviews and meta-analysis

**DOI:** 10.1097/MD.0000000000026116

**Published:** 2022-10-14

**Authors:** Xingchen Zhou, Zhizhen Lv, Shuangwei Hong, Huijie Hu, Yu Tian, Shuang Wu, Kaizheng Wang, Zicheng Wei, Lijiang Lv

**Affiliations:** a The Third Clinical Medical College of Zhejiang Chinese Medical University, Hangzhou, Zhejiang, China.

**Keywords:** acupuncture, AMSTAR-2, chronic prostatitis, GRADE, moxibustion, overview, PRISMA

## Abstract

Chronic prostatitis (CP) refers to a disease characterized by local pain and discomfort, urination discomfort, and quality of life. Acupuncture (ACU) and moxibustion are widely used in the treatment of CP, and the curative effect is satisfactory. Several systematic reviews (SRs) and meta-analyzes have reported the effectiveness of ACU and moxibustion in treating patients with CP. However, the evidence is not systematically integrated. This overview aims to integrate and evaluate the reliability of these SRs and the evidence generated from the ACU and moxibustion for CP meta-analysis.

**Methods:**

We will make a comprehensive retrieval in seven databases as following: Embase, Cochrane Library, Pubmed, Chinese databases SinoMed (previously called the Chinese Biomedical Database), Chinese National Knowledge Infrastructure (CNKI), Chinese Scientific Journals Database (VIP), and Wanfang Data (WF). The time is limited from the construction of the library to May 2021. We will use the Assessment of Multiple Systematic Reviews-2 (AMSTAR-2) tool to evaluate methodological quality. Preferred Reporting Items for Systematic Reviews and Meta-analysis Protocols (PRISMA-P) will be used in the report checklist to assess the quality of reports in the study. The GRADE will be used to evaluate the included SRs and meta-analysis. Our reviewers will conduct SRs, qualification evaluation, data extraction, methodological quality and evidence quality screening in pairs. The outcomes of interest include: NIH-Chronic Prostatitis Symptom Index (NIH-CPSI), effective rate, other CP symptom scales, EPS-WBC, and adverse events. Evidence will be combined based on patient subgroups and results where appropriate.

**Results:**

The results will be published in a peer-reviewed journal.

**Trial registration number:**

INPLASY202150018.

**Conclusion:**

This overview will provide comprehensive evidence of ACU and moxibustion for patients with CP.

## 1. Introduction

Chronic prostatitis (CP) has a high incidence and a low cure rate, which seriously affects the life and work of patients.^[1–3]^ The clinical symptoms are mainly manifested in three significant aspects: local pain and discomfort, urinary discomfort, and decreased quality of life.^[4,5]^ The incidence of this disease in men reaches 8.2%.^[6]^ Patients account for 25% to 33% of outpatients in the urology department. The recurrence rate of CP is 25%, and 50%, about 50% of men have had symptoms of CP at different periods of their lives.^[7,8]^ Therefore, it is necessary to study the social problems caused by CP to improve the physical and mental health of patients.

Modern medicine believes that the cause of this disease is complex,^[9,10]^ which can be caused by pathogen infection, immune abnormalities, urine reflux, enhanced oxidative stress, urinary dysfunction, prostate stasis, mental and psychological factors, neuroendocrine factors, autoimmune factors, and allergic reactions. A variety of factors lead to the formation of a chronic pathogenic process through multiple pathogenic mechanisms. The mechanism is still unclear.^[11,12]^

Western medical treatment generally aims to improve symptoms, improve the quality of life, and promote the recovery of related functions.^[13]^ So far, there is no good treatment for non-inflammatory CP, and most treatments have not achieved good results. Furthermore, inflammatory CP treatment usually uses alpha-blockers, anti-inflammatory drugs, physical therapy, anxiolytics, and antidepressants.^[14,15]^ These treatments can indeed play a key role, but the side effects of the drugs are troublesome. Therefore, safer and more effective alternative therapies are urgently needed by patients, such as ACU and moxibustion, which are considered the best options by the general public.

CP belongs to the “gonorrhea” in Traditional Chinese medicine (TCM).^[16]^ Based on the TCM, the pathogenesis is closely related to Ren Channel, Foot Shaoyin Channel, Foot Taiyin Channel.^[17]^ This disease is mainly caused by improper diet, addiction to alcohol, wine, and sweetness, which produces damp heat, or due to external feelings of damp and heat, congregating in the lower coke; or due to phase fire, unsuccessful wishes, or tolerance but not diarrhea, kidney fire It does not disperse, the essence of the dislocation turns into white turbidity, or the intercourse is unclean, the damp heat invades from the essence tract, the damp heat stagnates, and the blood is blocked.^[18,19]^ The main essential syndrome types of CP are: damp-heat infusion syndrome, qi stagnation, and blood syndrome, liver-qi stagnation syndrome, kidney-yang deficiency syndrome, kidney-yin deficiency syndrome, and the compound syndrome type is damp-heat stagnation syndrome. In short, dampness, heat, silt, stagnation, and deficiency run through different stages of CP.^[20,21]^

ACU and moxibustion are suitable TCM treatment methods. CP treatment has a long history and long-term clinical practice. Studies believe that these therapies have unique clinical effects on CP.^[22,23]^ Studies have found that ACU and moxibustion can improve the blood circulation of the prostate and relieve the spasm of smooth muscles so that the patient’s local symptoms and systemic functions can be improved.^[24]^ Besides, these therapies can also speed up blood circulation, effectively reduce the secretion of inflammatory substances, promote the absorption of inflammatory exudates, and play a role in eliminating inflammation.^[25]^

Many randomized controlled trials (RCTs) have confirmed the efficacy of ACU and moxibustion in CP treatment.^[26,27]^ Many meta-analyses also show that ACU and moxibustion treatment has specific benefits for CP patients.^[28,29]^ However, no investigator has made an overview to evaluate the systematic review of ACU and moxibustion on CP. Therefore, this study assessed and summarized the clinical research literature on AUC and moxibustion in CP at home and abroad and provided evidence-based medicine for clinical practice.

## 2. Methods

### 2.1. Study registration

This protocol was designed based on the overview methodological guidelines provided in the Cochrane System Intervention Review Manual.^[30]^ It is registered on the International Prospective Register of Systematic Reviews (registration number INPLASY202150018; https://inplasy.com/inplasy-2021-5-0018/.)

### 2.2. Inclusion and exclusion criteria

PICOS will be applied. Including Population, Intervention, Comparison, Outcome, and Study.

#### 2.2..1. Type of study.

It only includes SRs and meta-analysis of RCTs on ACU and moxibustion in CP patients. The language will be limited to Chinese and English.

#### 2.2..2. Type of participants.

A systematic review of people diagnosed with CP will be included. Regardless of gender, race, occupation, education, nationality, etiology, and severity.

#### 2.2..3. Type of interventions.

ACU treatments include moxibustion, catgut embedding, electro-acupuncture, transcutaneous electrical acupoint stimulation, auricular acupuncture, scalp acupuncture, warm needling, manual acupuncture, acupoint injection, regardless of needling techniques and stimulation method.

#### 2.2..4. Type of comparator (s)/control.

The control group’s treatment is not limited, including no treatment, placebo, or any control considered for comparison in a single systematic review.

#### 2.2..5. Types of outcome measurements.

##### 2.2..5..1. Primary outcomes.

NIH-Chronic Prostatitis Symptom Index (NIH-CPSI).

##### 2.2..5..2. Secondary outcomes.

Secondary outcomes mainly include the following aspects:

1. Effective rate.
2. Other CP symptom scales.
3. EPS-WBC.
4. Adverse events.

#### 2.2..6. Study design.

An SR that contains more than one RCTs. There is no systematic review, no separate abstract of RCTs data, and abstracts that do not have enough data will be excluded.

### 2.3. Search methods for identification of studies

We searched three foreign electronic databases (Cochrane Library, Embase, Pubmed) and four Chinese electronic databases (China National Knowledge Infrastructure (CNKI), WangFang Database, Chinese Biomedical Literature Database (CBM) and Chinese Scientific Journal Database (VIP) to collect potential systematic reviews (SRs) from their inceptions to May 2021. The language of publication is limited to Chinese or English. The following search terms will be used: CP, abacterial prostatitis, prostatodynia, prostatalgia, prostate pain, moxibustion, thunder fire miraculous moxa roll, thunder fire moxibustion, taiyi miraculous moxa roll, suspended moxibustion,mild moxibustion, needle warming moxibustion, systematic review, meta-analysis, et al. A draft search strategy using Pubmed, one of the planned electronic databases to be searched, is presented in Table 1.

**Table 1:** Search strategy (PubMed).

Number	Search terms
#1	MeSH: “Acupuncture” OR “Acupuncture Therapy” OR “Acupuncture, Ear”
#2	Ti/Ab: “acupuncture” OR “acupoint” OR “acupoint injection” OR “acupuncture therapy” OR “electroacupuncture” OR “auriculotherapy” OR “acupoint catgut embedding” OR “moxibustion” “moxa” OR “needle” OR “warm needle” OR “temperature needle” OR “needle”
#3	#1 OR #2
#4	MeSH: “Moxibustion”
#5	Ti/Ab: “Thunder-fire miraculous moxa roll” OR “Thunder fire moxibustion” OR “taiyi miraculous moxa roll” “suspended moxibustion” OR “mild moxibustion” OR “needle warming moxibustion”
#6	#4 OR #5
#7	MeSH: “ chronic prostatitis ”
#8	Ti/Ab: “ abacterial prostatitis ” OR “ prostatodynia ” OR “ prostatalgia ” OR “ prostate pain ”
#9	#7 OR #8
#10	MeSH: “Systematic Reviews as Topic” OR “Systematic Review” OR “Meta-Analysis” OR “Meta-Analysis as Topic” OR “NetworkMeta-Analysis”
#11	Ti/Ab: “Systematic Review” OR “Meta-Analysis” OR “Network Meta-Analysis”
#12	#10 OR #11
#13	#3 AND #6 AND #9 AND #12

Ab = abstract, Ti = title.

### 2.4. Studies selection

**Figure 1.**
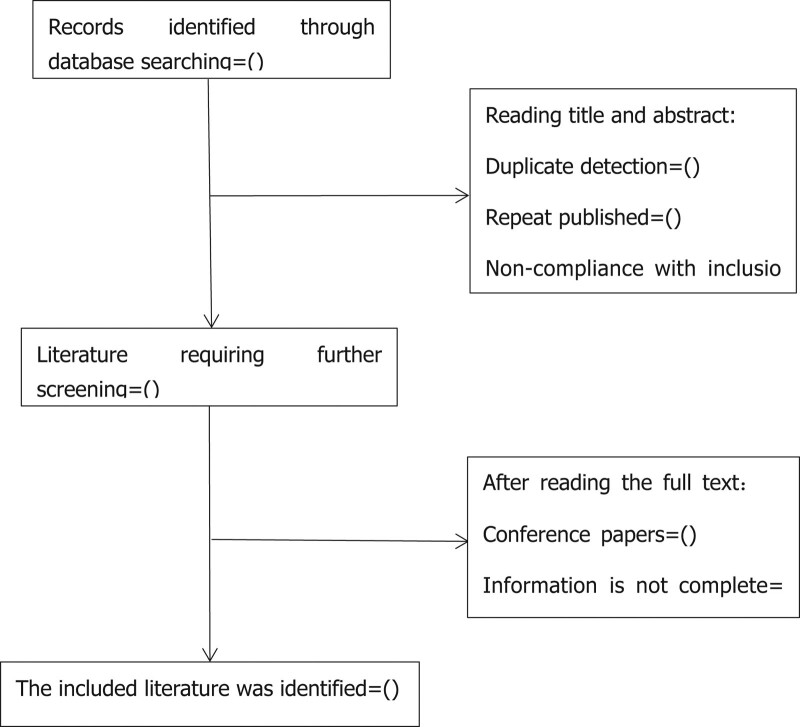
Flowchart of literature selection.

Studies will be identified using NoteExpress 3.2.0 (NoteExpress, Beijing Aegean Sea LezhiTechnologyCo., Ltd.). After the initial removal of duplicate studies, two reviewers (GMH and ZRL) will independently screen titles and abstracts based on the eligibility criteria. Full-text studies will be retrieved for all potentially includable SRs or SR protocols. If studies contain insufficient information to make a decision about eligibility, XCZ will try to contact authors of the original reports to obtain further details. During the procedure, disagreements will be resolved by discussion or consensus with the third reviewer (DSW). Study selection will be performed in accordance with the Preferred Reporting Items for Systematic Reviews and Meta-Analyses (PRISMA) flowchart (Fig. 1).

### 2.5. Data extraction

Two researchers (QJM and YNY) extracted literature information based on inclusion and exclusion criteria, including the following:

1. Study characteristics: author, year, study design, sample size and follow-up time;
2. Patient characteristics: age, sex, and type of SP;
3. Intervention: intervention measures in the experimental group, intervention measures in the control group);
4. Outcome of the study: Two researchers (XCZ and SSZ) cross-checked the extraction results of the extracted documents. If there is any difference, you should consult a third party (ZHC) to resolve.

### 2.6. Evaluate the methodological quality of included studies

Two reviewers (XCZ and ZHC) will use the Assessment of Multiple Systematic Reviews 2 (AMSTAR-2) measurement tool to independently assess each SRs methodological quality that meets the inclusion criteria.^[29]^ This is most commonly used to assess the quality of SRs included in overviews. AMSTAR-2 is an update of AMSTAR, which can be used to appraise SRs of both randomized and non-RCTs. AMSTAR-2 includes 16 items, with each of the 16 criteria given a rating of “yes” (definitely done), “no” (definitely not done), “cannot report” (unclear if completed), or “not applicable” based on the information provided by the systematic review, when the standards are met, the evaluator will evaluate the evaluation. Disagreements will be resolved through discussions between them and arbitrated by the third general author (DSW) if necessary.

### 2.7. Evaluation of the reporting quality of the included studies

The two authors of the overview (YNY and QJM) will independently evaluate the reports’ quality in each review to assess whether they meet the criteria specified in Preferred Reporting Items for Systematic Reviews and Meta-analysis Protocols (PRISMA-P).^[30]^ If there are any differences, they will be resolved through discussion between them and arbitrated by the third general author (SSZ) if necessary.

### 2.8. Evaluation of the evidence quality of the included studies

The quality of evidence of the included SRs was assessed by the Grading of Recommendations Assessment, Development and Evaluation (GRADE) approach.^[31]^ This tool aims to assess the quality of evidence for each outcome indicator in the study. The two authors (ZHC and XCZ) will independently evaluate the evidence of the results and should describe in detail the degradation or upgrade factors that affect the quality of the evidence to ensure the reliability and transparency of the results. Any disagreements will be resolved through discussion by two authors. The overall quality of evidence was judged as “high,” “moderate,” “low,” or “very low.”

### 2.9. Dealing with lost data

If there is no specific data or insufficient data in the published SRs, the author will be contacted by email or phone to provide the necessary information. If we cannot obtain enough data, the data will be discarded. The analysis will be based on the available data and the potential impact of missing data will be discussed.

### 2.10. Synthesis of data

Before data synthesis, the included SRs and meta-analysis should be considered. For different situations, different measures will be taken for overlapping basic research: whether the basic research completely overlaps, the comment with the highest quality will be selected. If the main research partially overlaps, when the lower quality reviews include more than one-third of the new research. If the basic research does not overlap, the two comments will remain. The quality of the review will be fully assessed Use ROBIS and AMSTAR-2. Besides, RevMan5.3.5 software will be used to calculate the standardized effect. The random-effects model (*I*^2^ ≥ 50%) or fixed-effects model (*I*^2^ < 50%) will be selected according to the heterogeneity levels of the included SRs and meta-analyses. If the *I*^2^ value is higher than 75%, the clinical or methodological heterogeneity will be explored through discussion with the review team. When the meta-analysis is not possible, a narrative analysis will be performed. Indirect comparisons of different ACU and moxibustion therapies will also be conducted using relative effectiveness outcomes including relative sensitivity and relative specificity.

## 3. Discussion

CP has a long course and recurrent symptoms, which can easily cause mental stress and psychological problems in patients. ACU and moxibustion, as practical techniques of TCM, have been accepted for CP in China. However, due to the lack of rigorous review evidence for ACU and moxibustion treatment, clinicians cannot choose the best method. As a result, patients with CP are prone to delays. Therefore, the results of this overview will provide accurate and reliable research evidence for the treatment of CP with ACU and moxibustion.

The study also has some defects as follows: low quality of original researches, the possible occurrence of false positive or false negative results, various duration of disease, frequency of intervention, language restriction, etc. All of these will lead to some bias and influence the results of evaluation results, ultimately affecting this study’s reliability.

## Author contributions

**Conceptualization:** Xingchen Zhou, Zhenhai Chi.

**Data curation:** Desheng Wu, Guomin Huang, Shuisheng Zhou, Qiangjian Mao, Yanan Yang, Zhenhai Chi, Ziru Li.

**Formal analysis:** Xingchen Zhou, Zhenhai Chi.

**Investigation:** Xingchen Zhou, Zhenhai Chi.

**Methodology:** Guomin Huang, Yanan Yang, Zhenhai Chi, Ziru Li.

**Software:** Shuisheng Zhou, Xingchen Zhou.

**Supervision:** Xingchen Zhou, Zhenhai Chi.

**Writing – original draft:** Qiangjian Mao, Xingchen Zhou, Zhenhai Chi.

**Writing – review & editing:** Desheng Wu, Xingchen Zhou, Zhenhai Chi.
